# Voluntary male medical circumcision of pre-school-aged boys in primary care

**DOI:** 10.4102/phcfm.v17i1.5039

**Published:** 2025-11-04

**Authors:** Norman D. Goldstuck, Peter S. Millard

**Affiliations:** 1 Department of Obstetrics and Gynaecology, Faculty of Medicine, Stellenbosch University, Cape Town, South Africa; 2 Department of Medicine, Faculty of Osteopathic Medicine, University of New England, Biddeford, United States of America

**Keywords:** HIV prevention, minimally invasive surgery, circumcision, Voluntary Medical Male Circumcision, VMMC, instrument-assisted circumcision, paediatric surgery, surgical instruments, primary care, Unicirc

## Abstract

Voluntary medical male circumcision (VMMC) is a priority human immunodeficiency virus (HIV) preventive intervention. Challenges in funding national VMMC programmes mandate us to adopt new methods to provide circumcisions in a primary care setting. This study aims to test the practicality of instrument-assisted circumcision in primary care in pre-school-age boys. The study setting was one primary care centre in Western Cape, South Africa. The methodology adopted is prospective case series of minimally invasive voluntary circumcision using the Unicirc instrument in boys less than 6 years of age. We circumcised 221 healthy boys using the Unicirc instrument, using a combination of topical anaesthetic, subcutaneous local anaesthetic and intramuscular ketamine. There were 5 (2.3%) mild complications and 48 (21.7%) had mucosal swelling as a result of lysis of physiological phimosis. All boys were fully healed at 2 weeks and all caregivers were highly satisfied. Using a circumcision instrument in primary care simplifies circumcision in pre-school-age boys and has a low rate of adverse events. This study demonstrates that a new model of circumcision in primary care may enhance national VMMC programmes.

## Background

Along with perinatal prophylaxis, antiretroviral treatment and pre-exposure prophylaxis (PREP), male circumcision comprises a crucial role in human immunodeficiency virus (HIV) prevention efforts in southern Africa.^1^ Since the implementation of World Health Organization (WHO) and Joint United Nations Programme on HIV/AIDS (UNAIDS) voluntary medical male circumcision (VMMC) programmes in 2008, 37 million VMMCs have been performed in 15 high priority sub-Saharan countries, preventing one million new HIV infections.^2^ The recent reduction in United States (US) government funding demands cost-effective local VMMC programmes.

In countries with a high prevalence of HIV, circumcision prior to the debut of sexual activity is an effective and long-term preventive intervention. The neonatal period is an ideal time to circumcise boys, because (unlike boys of an age greater than 1 month), suturing the line of excision is not needed and the risk of bleeding is very low. The procedure is either performed in hospital prior to discharge of the newborn or in the home as part of a religious ceremony.

Given the simplicity of the procedure when performed with a surgical instrument (e.g., the Gomco clamp or the Mogen clamp), the frequency of adverse events is 4 per 1000 procedures for early infant circumcision. However, this number increases 10- to 20-fold in older boys who have historically been circumcised using open surgical methods by surgeons.^3^

**FIGURE 1:**
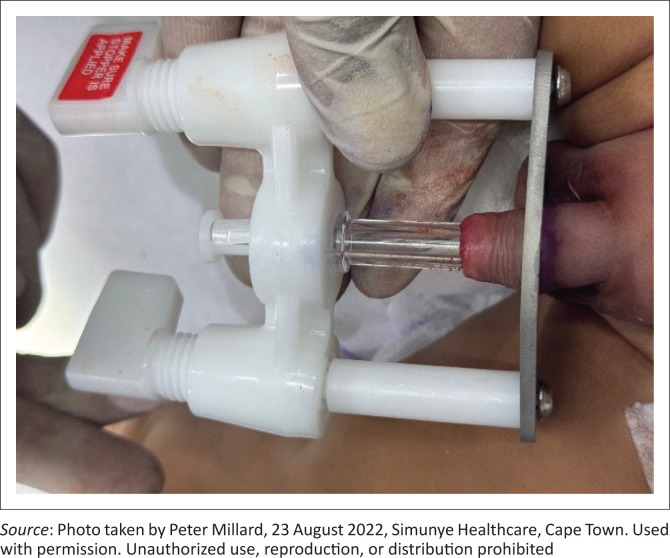
Unicirc after placement in a young boy.

Based on the Gomco clamp, the Unicirc device is a commercially available, single-use-only, disposable plastic and metal instrument developed and licensed in South Africa (Figure 1). Simunye Primary Health Care has been using the Unicirc to perform VMMCs in men and boys of all ages for over a decade.^4^ Unicirc enables circumcision from childhood onwards, and is available in seven different sizes, depending on age and glans diameter.

## Research methods and design

This is a prospective case series of circumcisions among boys from the newborn period less than 6 years of age in a single primary care practice in Mitchell’s Plain, South Africa. All circumcisions were performed by family physicians.

### Analgesia

Our protocol for analgesia and sedation was based on best practices in this age group.

Healthy boys were fasted for 2 h prior to the procedure, and those with upper respiratory or other infections were excluded. We measured oxygen saturation continuously and had suction available.

### Surgical procedure

The method of using Unicirc included freeing of adhesions and performing a dorsal slit where indicated and has been described in detail previously.^5,6^ We used cyanoacrylate tissue adhesive to prevent the excision line from separating and bleeding.

### Ethical considerations

Ethical approval to conduct this study was obtained from The South African Medical Association Research Ethics Committee (SAMAREC). Parents or guardians provided written informed consent for the surgical procedure and reporting of outcome data. Permission to photograph was obtained separately. All data were anonymised and kept confidential. The SAMAREC approved Unicirc for Phase IV (post-licensing) use (06 March 2014).

## Results

The circumcisions were performed on all 221 boys consecutively from June 2020 to August 2023.

The boys’ age and reasons for circumcision are shown in Table 1. Median age was 15 months and the median weight was 11 kg.

**TABLE 1:** Age distribution and reason for circumcision (*N* = 221).

Characteristic	*n*	%
**Age (years)**		
< 1	37	16.7
1–3	135	61.1
4–5	49	22.2
**Reason for circumcision**		
Religion	139	62.9
Hygiene	66	29.9
Health or disease prevention	16	7.2

All boys required foreskin dilatation; overall 40% required a dorsal slit in order to insert the transparent bell over the glans.

### Anaesthesia

Prior to any local anaesthetic injection, we used Emla (lidocaine and prilocaine) topical cream at the injection site. The main analgesic modality was a penile ring block, which is a simple subcutaneous injection of lidocaine and bupivacaine mixture at the base of the penis.

Sedation is needed, especially in the 1 year to 3 year age group, and intramuscular ketamine was the agent of choice. It causes no respiratory depression or suppression of the gag reflex, is effective for the time required with a single intramuscular dose of 5 mg/kg body weight and provides post-operative analgesia. Overall, 81% of the boys required ketamine sedation.

All boys were fully healed within 14 days. Caregivers uniformly reported that cosmetic results were excellent.

### Adverse events

All circumcisions were completed within 15 min. No serious adverse events occurred. Three boys had post-operative adhesions, which were released under topical anaesthetic and two boys had mild bleeding, treated with additional tissue adhesive. As expected, 48 boys (21.7%) had mucosal swelling as a result of lysis of physiological adhesions. There were no serious adverse events.

## Discussion

Given the recent reduction of external funding, it is imperative for areas with high HIV prevalence to develop cost-effective domestic VMMC programmes. New techniques for circumcising boys in the primary care realm, where preventive care is most effectively delivered, are a promising avenue.

The newborn period is the ideal time for circumcision. It is often performed by house officers with minimal training and few complications. After that, the risk of adverse events goes up substantially and young boys do not cooperate. However, many mothers want their boys circumcised. Generally, circumcision has been carried out in the pre-school age group by specialists under general anaesthesia.

The open surgical method for post-neonatal circumcision is now outdated. This study shows for the first time that circumcision can be safely performed in a primary care setting in this age group using simple intramuscular sedation and a novel surgical instrument developed in Africa.

Unicirc has features that make it more attractive than other options. It is a commercially available single-use, pre-sterilised, disposable instrument, which prevents potential cross contamination. The disadvantages of devices such as the Shang Ring are that they require a second procedure and specialised instruments for removal after 1 week, and delayed healing by secondary intention may result in disease transmission.^7^

Training to do open surgical circumcisions, on the other hand, is labour intensive and time consuming. Complication rates are high and cosmetic results are less than ideal.

We have previously trained nurses to perform Unicirc circumcisions,^8^ and this study shows for the first time that it is also possible to perform it safely in the critical pre-school age group. With the right instruments, primary care providers can and should offer this service.

Primary care is the ideal venue for performing circumcision in all ages and, taking a lesson from immunisation programmes, ‘missed opportunities’ can be prevented by providing this service where men and boys come for care.
